# A bicarbonate-rich liquid condensed phase in non-saturated solutions in the absence of divalent cations

**DOI:** 10.3389/fbioe.2024.1382047

**Published:** 2024-04-30

**Authors:** Mark A. Bewernitz, Matthew Ginder-Vogel, Stephan E. Wolf, Jong Seto, Brent R. Constantz

**Affiliations:** ^1^ Physical Sciences Department, College of Arts and Sciences, Embry-Riddle Aeronautical University, Daytona Beach, FL, United States; ^2^ Environmental Chemistry and Technology Program, Department of Civil and Environmental Engineering, University of Wisconsin—Madison, Madison, WI, United States; ^3^ Department of Materials Science and Engineering, Institute of Glass and Ceramics, Friedrich-Alexander-University Erlangen-Nürnberg (FAU), Erlangen, Germany; ^4^ Interdisciplinary Center for Functional Particle Systems (FPS), Friedrich-Alexander University Erlangen-Nürnberg (FAU), Erlangen, Germany; ^5^ Center for Biological Physics and School for Engineering of Matter, Transport, and Energy, Arizona State University, Tempe, AZ, United States; ^6^ Blue Planet, Ltd., Los Gatos, CA, United States

**Keywords:** liquid condensed phase, nanoparticle tracking analysis, bicarbonate, biomineralization, pair distribution function

## Abstract

Bicarbonate (HCO_3_^−^) and sodium (Na^+^)-containing solutions contain droplets of a separate, bicarbonate-rich liquid condensed phase (LCP) that have higher concentrations of HCO_3_^−^ relative to the bulk solution in which they reside. The existence and composition of the LCP droplets has been investigated by nanoparticle tracking analysis, nuclear magnetic resonance spectroscopy, refractive index measurements and X-ray pair distribution function analysis. The bicarbonate-rich LCP species is a previously unaccounted-for, ionic phenomenon which occurs even in solutions with solely monovalent cations. Its existence requires re-evaluation of models used to describe and model aqueous solution physicochemistry, especially those used to describe and model carbonate mineral formation.

## Introduction

In spite of the ubiquity of calcium carbonate and its deep involvement in biological, geological and industrial processes, the mechanisms by which inorganic carbonates separate from the mother solution during the process of solid nucleation and precipitation are still a matter of ongoing debate. Besides the classical pathway toward carbonate mineral nucleation and growth, which is driven by nucleation and ion-mediated growth of post-critical clusters, various nonclassical pathways were put forward and identified in recent years. These nonclassical pathways are driven by the formation of entities containing multiple Ca^2+^ and CO_3_^2-^ ions (e.g., complexes, clusters, polymers or colloids) rather than individual attaching ions or molecules (Niederberger and Colfen, 2006; De Yoreo et al., 2015). The formation of calcium carbonate, in the absence of inhibitors heterogeneous surfaces or rapid (crash) precipitation, follows a non-classical multi-step process to solid nucleation in which, as a first step, a calcium and carbonate ion-rich liquid condensed phase (LCP) phase-separates from the bulk solution (Gower and Odom, 2000; Rieger et al., 2007; Wolf et al., 2008; Wolf et al., 2015) followed by further steps directing solidification, nucleation, and growth of calcium carbonate mineral. Polyelectrolytes and small molecular species, such as those found in seawater and biological systems, impact the stability and abundance of the LCP (Gower, 2008; Wolf et al., 2008; Vekilov, 2010; Wolf et al., 2011a; Gebauer and Coelfen, 2011; Schenk et al., 2012; Wolf et al., 2012; Wallace et al., 2013). Mechanisms for liquid-liquid separation and demixing into a LCP, such as calcium carbonate pre-nucleation clustering leading to a calcium carbonate DOLLOP (dynamically ordered liquid-like oligomer phase), have been proposed which are driven largely by divalent cation (Ca^2+^)-to divalent anion (CO_3_^2-^) interaction as well as aqueous solvation properties of the ions. The hydrated ionic complexes formed are described as solute phenomena, limited through entropic effects, in size and duration. However, it has not yet been experimentally established how these phenomena may be involved in the formation of relatively long-lived, relatively large scale liquid droplets observed in LCP formation. A recent molecular modelling study supports the potential spinodal decomposition of the homogenous solution containing calcium and carbonate ions (Wallace et al., 2013); it is also possible that pre-critical complexes cluster into a separate phase and therefore serve as building blocks of the LCP due to weak interactions and hydrogen bonding (Wolf et al., 2008; Wolf et al., 2011b; Wolf et al., 2017) which may give rise polyamorphicity of calcium carbonate (Cartwright et al., 2012; Gebauer et al., 2014).

These, and many studies are conducted at an elevated, less biologically relevant pH (i.e., pH > 10) where the speciation of the bicarbonate/carbonate system favors the carbonate ion. These elevated pH values are only relevant to a few applications, e.g., industrial scale formation. However, in biological or marine environments the pH is often closer to neutral and thus dissolved carbonate speciation is dominated by the bicarbonate ion rather than the carbonate ion (Supplementary Figure S1). Recent studies indicate that at near neutral pH, LCP formation is eased, its life time is on the order of several minutes or longer (Wolf et al., 2008), and that bicarbonate–not carbonate ions–condense into a bicarbonate-rich LCP which serve as the condensation node for primary, solid, nucleation (Bewernitz et al., 2012a). The metastability of the bicarbonate-rich LCP in the presence of divalent cations (e.g., Mg^2+^, Ca^2+^) is proposed to be due to the affinity of divalent cations to associate with anions as well as the requirement of the resulting bicarbonate-rich LCP to expel hydronium ions before transforming into solid amorphous or crystalline calcium carbonate (Wolf et al., 2011a). The mechanism by which this liquid-condensed precursor phase actually forms, is unclear; both the attraction of ions through strong divalent positive-to-negative attractions and the role of water solvation properties, through binodal and spinodal separation mechanisms, have been proposed and are being actively debated (Faatz et al., 2005; Rieger et al., 2007; Dorvee and Veis, 2013; Wallace et al., 2013; Sebastiani et al., 2016). Similar experiments performed elsewhere using photo-decarboxylation reactions of a Ketoprofen compound demonstrate the ability to produce stable HCO3^-^ moieties (Menichetti et al., 2021). In many of these discussions, the LCP phase is described as a transient precursor, driven by ion charge attraction (between Ca^2+^ and CO_3_^2-^ for the calcium carbonate system), which exists only briefly before formation of solid, stable, CaCO_3_ nuclei.

Here, we report that a bicarbonate-rich liquid-condensed phase exists in undersaturated solutions of simple bicarbonate salts, in the absence of divalent ions such as Ca^2+^. We provide evidence that the formation of bicarbonate-rich LCP is not necessarily specific to calcium carbonate systems and exists independent from supersaturation levels of the related monovalent inorganic bicarbonate solid-phases. The bicarbonate-rich LCP appears to be in equilibrium with the bulk solution and thus not a transient, precipitating, phase *enroute* to formation of a solid phase, but is present as a stable heterogeneous distribution of bicarbonate ions, as well as other ions, within the bulk phase. Our findings suggest that bicarbonate ions in solution exhibit a fundamental behavior of macro-scale association, even in the absence of divalent positive-to-negative ionic attraction. This observation provides evidence that the solvation properties of bicarbonate ions in water play a significant role in the formation of liquid condensed phases. These results have fundamental ramifications in fields including oceanography, biomineralization, environmental sciences, and nucleation theory (Lee et al., 2010).

**FIGURE 1:**
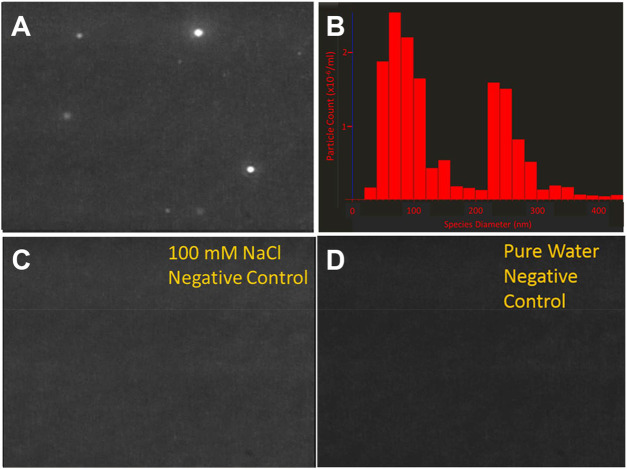
Images of LCP droplets in various solutions at 22°C, as determined by nanoparticle tracking analysis (NTA). Nanoparticle tracking analysis (NTA) detect the presence of scattering events within NaHCO_3_/NaCl solutions in the absence of saturation conditions and divalent ions, such as Ca^2+^ or Mg^2+^. The solutions were filtered through 20 nm diameter syringe filters prior to analysis. **(A)** A 50 mM NaHCO_3_, 50 mM NaCl solution contains scattering events **(B)** The diameter of scattering events observed in 1A are on the order of 50–300 nm. This is much larger than the 20 nm pores through which the solution was filtered **(C)** 100 mM NaCl, which was also filtered does not contain scattering events **(D)** Distilled, deionized water, which was also filtered, does not contain scattering events. This data suggests that bicarbonate ions are essential in the formation of the relatively large scattering species. This species is differentiated from the mother solution by density and/or salinity which would lead to a refractive index contrast with respect to the mother solution.

Nanoparticle tracking analysis (NTA) is a light scattering technique which identifies colloids and other scattering events in a variety of solutions. NTA is preferred over dynamic light scattering in characterizing the size distribution of LCP species due to its sensitivity, high spatial resolution (Filipe et al., 2010), and its ability to detect LCP species, demonstrated in a previous study (Bewernitz et al., 2012a). In contrast to dynamic light scattering, NTA does not fit a predetermined scattering size distribution to an ensemble of collected scattering events, rather it tracks a projection of individual scattering events (detected as intensities contrasts from the background), and assigns a diameter to the event based on the recorded Brownian motion. Therefore, NTA requires a sample to be several orders of magnitude more dilute in scattering events than dynamic light scattering. We observe distinct scattering events while investigating pure solutions of monovalent bicarbonate (e.g., sodium bicarbonate) prepared at low, under-saturated concentrations. Still-shots of the scattering events and the size distribution of scattering events are shown in Figure 1A. In a solution containing 50 mM NaHCO_3_ and 50 mM NaCl (Figure 1A) several dozens of scattering events are observed at any given moment ranging in size from 10 nm (lower limit of detection) to 350 nm in diameter. Critically, the sample in Figure 1A is undersaturated with respect to sodium chloride and sodium bicarbonate, contains only monovalent inorganic anions with no divalent cations and has been filtered through a 0.020 μm syringe filter, prior to analysis. These conditions represent a sodium bicarbonate solubility product of ∼5% of saturation with respect to nahcolite, and << 1% of saturation with respect to solid sodium chloride, effectively eliminating the possibility that the detected phase is precipitated mineral. Scattering is not observed in the absence of bicarbonate in the sodium chloride control (Figure 1C) or the pure water control (Figure 1D). Both of these samples were also filtered through a 0.020 um syringe filter suggesting that the bicarbonate ion is a required component for formation of phases responsible for the scattering events in solution.

**FIGURE 2:**
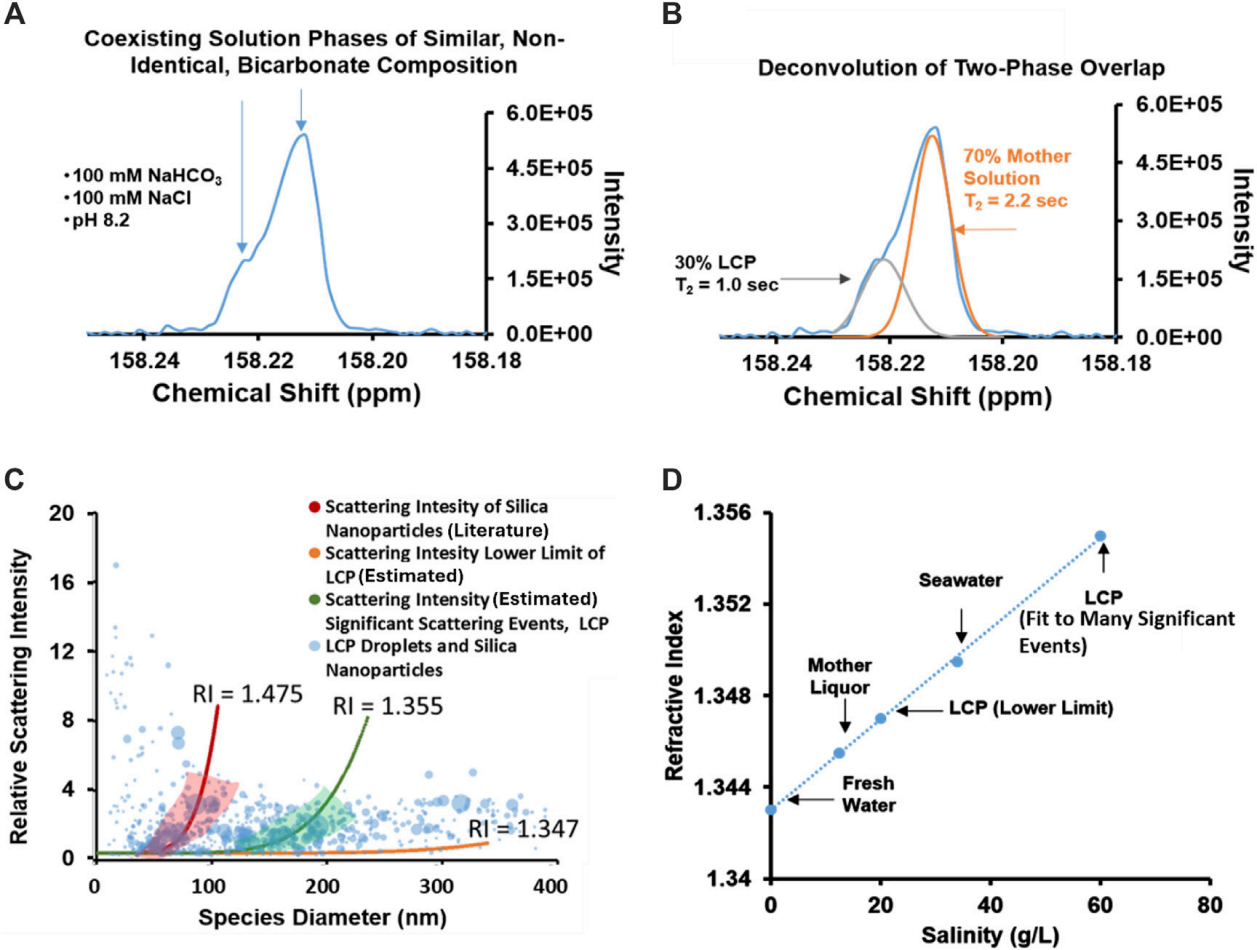
Results from nuclear magnetic resonance spectroscopy and nanoparticle tracking analysis indicate that the bicarbonate-rich LCP behaves as a two-phase, solution-state system. **(A)** ^13^C NMR data of a 100 mM NaHCO_3_ (100% ^13^C-enriched), 100 mM NaCl solution. The shoulder at 158.22 ppm is attributable to the presence of a distinct bicarbonate-containing phase; an LCP (Bewernitz et al., 2012a). Differences in chemical shift may be due to differences between the tumbling mobility and magnetic nuclear susceptibility of bicarbonates in the LCP as compared to bicarbonates in the mother solution phase. **(B)** Deconvolution of the overlapping peaks in the ^13^C NMR spectrum in **(A)** suggests that approximately 30% of the inorganic carbon has participated in LCP over the 6.35 s acquisition time. As much as 70% remained in the mother liquor phase during acquisition. **(C)** Qualitative analysis of relative light scattering intensities of LCP droplets in a 100 mM NaHCO_3_ (naturally occurring ^12^C prevalence), 100 mM NaCl solution, similar to the solution analyzed by NMR in Figures 2A,B. The bicarbonate-rich LCP appears to have refractive indices consistent with that of salt water. The range of RI values qualitatively assigned for the bicarbonate-rich LCP droplets is slightly larger than the RI of fresh water alone, consistent with saltwater solutions **(D)**. The linear relationship between salinity (g/L) and the RI based on data from literature (Quan and Fry, 1995). According to the relationship, LCP in this solution appears to have a lower-limit salinity of ∼20 g/L and upper limit salinity of ∼60 g/L, assuming salinity is the main driver of R.I. changes. Both values are more concentrated than the mother solution from which they are derived (the background which is the relative reference) suggesting that the scattering phase is concentrated in ionic solutes.

Nuclear Magnetic Resonance (NMR) is a useful tool for investigating bicarbonate/carbonate nucleation behavior (Gebauer et al., 2010; Bewernitz et al., 2012a) and is utilized to further characterize the species observed in the sodium bicarbonate/sodium chloride solutions seen in Figure 1. A 100 mM NaHCO_3_, 100 mM NaCl solution (1:1 bicarbonate-to-chloride anion ratio), similar to the 1:1 bicarbonate-to-chloride ratio present in the solution analyzed in Figure 1A, was characterized using 1D, transverse relaxation measurements (see Figures 2A, B). Information regarding pulse sequence, sample preparation, and data analysis, are detailed in the associated content. A chemical shift standard was not added to the solution to ensure electrolyte behavior is un-altered. The 1D, ^13^C NMR spectrum in Figure 2A shows an overlapping doublet (a main peak with a “shoulder”) which is consistent with the presence of two similar, yet non-identical, aqueous bicarbonate-dominated chemical equilibrium environments, e.g., HCO_3_^−^ interacting in/with a minority phase and HCO_3_^−^ interacting in/with the majority phase. Deconvolution of the overlapping peaks allows for the estimation of the fraction of bicarbonate ion participating in each environment due to the additive nature of ^13^C NMR, under these conditions. A still shot scattering image obtained with NTA (Supplementary Figure S2) of scattering events and an illustration of the deconvolution, attenuation and T_2_ determination of the two distinct, overlapping peaks (Supplementary Figure S3) are available in the associated content. Deconvolution of the signal in Figure 2A (Figure 2B) suggests that over the course of the 6-s used to acquire the data, approximately 30% of the inorganic carbon (bicarbonate ion) signal in the sample originates in a minor chemical environment that is distinct from inorganic carbon signal in the majority chemical environment. The T_2_ relaxation of the deconvoluted peaks was obtained through a Carr-Purcell-Meiboom-Gill (CPMG) method and is 1.0 s and 2.2 s for the minority bicarbonate phase and majority bicarbonate phase, respectively. The shorter, 1.0 s, relaxation time is consistent with bicarbonate ions participating in a more viscous, less mobile, and/or more concentrated environment than the bicarbonate ions in the mother solution with a larger relaxation time of 2.2 s. The presence of different HCO_3_^−^ ion T_2_ relaxation times for the same solute within a solution, re-enforces the notion that the two peaks are due to them residing in two distinct environments of liquid character. The timeframes of the T_2_ relaxation times assigned to the ^13^C in the bicarbonate-rich LCP and the mother solution (1.0 and 2.2 s, respectively) are consistent with solution-state, solvated ions. This provides evidence that bicarbonate ions are existing in a so-called bicarbonate-rich LCP comprised of solvated bicarbonate ions, in a distinct aqueous phase from that of bicarbonate ions in the mother solution. This is consistent with a recent study which defined bicarbonate-rich liquid condensed phase by means of similar NTA and NMR characterizations of the bicarbonate ion in the presence of calcium ion at concentrations that exceeded saturation with respect to solid CaCO_3_ (Bewernitz et al., 2012a). The data presented here suggests that the similar bicarbonate rich LCP may exist in solutions that are undersaturated at equilibrium and undersaturated with respect to any known solid mineral.

In addition to identifying particle size and number of particles (Figure 1), NTA provides a method of estimating the refractive index (RI) of particles (Filipe et al., 2010; Bewernitz et al., 2012a). NTA can qualitatively estimate the relative RI value of particles (e.g., LCP droplets) vs. a mother solution by comparison of the intensity vs. size profile of nanoparticles with a known size and RI (Figure 2C). A detailed description of the method for quantifying RI and a standard silica curve for RI fitting is available in the associated content and in Supplementary Figure S4 of the associated content, respectively. The area of the presented data point is proportional to the relevancy of the measurement and is based on the number of recorded random walks. SiO_2_ nanoparticles were used as a calibration standard (see associated content Supplementary Figure S5). Many qualitatively relevant, overlapping, scattering events (large data point in green shaded region) display a refractive index of approximately 1.355 Additional data points outside of the regime are encompassed by a lower limit RI of ∼1.347 (black line). The RI values encompassed in both of these regimes fall between 1.347 and 1.355, which is consistent with salt-water refractive indices but not with solid NaCl, NaHCO_3_, or Na_2_CO_3._ The salinity of the bicarbonate-rich LCP may be estimated by comparison of the measured RI to literature RI value of ion-rich salt water (Figure 2D) (Quan and Fry, 1995). According to this approach, the bicarbonate-rich LCP have an R.I. consistent with a salinity of between 20 and 60 g/L within a mother solution which has a salinity of 15 g/L (Figure 2D). This is consistent with reports describing LCP as a condensed solution state of bicarbonate and other ions (Bewernitz et al., 2012b) and to the best of our knowledge, represents the first estimation of the salinity of bicarbonate-rich LCP droplets, through direct observation with NTA.

**FIGURE 3:**
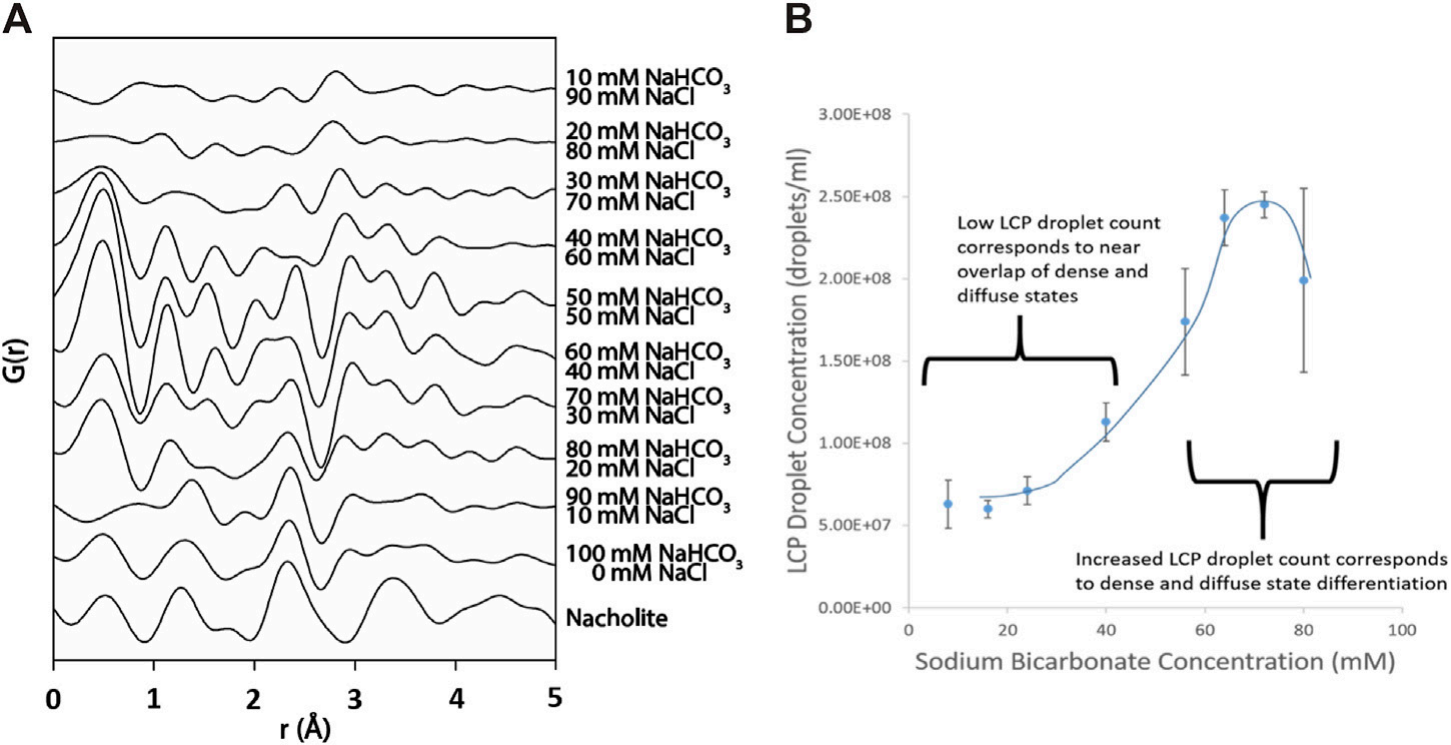
The formation of large species in the presence of undersaturated concentrations of sodium bicarbonate was investigated in sodium bicarbonate solutions of varying concentration using pair distribution function analysis (PDF) and nanoparticle tracking analysis (NTA). **(A)** PDF analysis demonstrating that above a sodium bicarbonate concentration of 30 mM, G_(Na,O1)_/G_(O,O)_, G_(Na,C)_, and G_(Na, O2)_ scattering features emerge at 2.4 and 3.25 Å and 3.8 Å, respectively. This suggests that there is a sodium-bicarbonate ion interaction, dissimilar from the shorter sodium-bicarbonate interaction seen in nahcolite, which is increasing with increasing sodium bicarbonate. **(B)** The concentration of bicarbonate-rich LCP droplets vs. the concentration of sodium bicarbonate in the sodium bicarbonate/sodium chloride solution. At approximately 30–40 mM NaHCO_3_ (70–60 mM NaCl, respectively) the LCP species count begins to increase significantly which corresponds directly with the conditions where the G_(Na,O)_ and G_(Na,C)_ association appears.

Although NTA and NMR data do not provide a direct measurement interatomic distance between the constituent atoms, they both indicate a fundamental shift in the chemical environment surrounding HCO_3_^−^ and a slight change in density as a result of bicarbonate-rich LCP formation, which results in a fundamental changes in the structure of solvating water encompassing the ions as well as changes in Na^+^ and HCO_3_^−^ interactions during LCP formation. X-ray, atomic pair distribution function (PDF) analysis is a total scattering technique, which considers both Bragg and diffuse scattering. It probes not only local atomic structure but also intermediate and long-range order to a distance of 10s of angstroms (Egami and Billinge, 2003). This makes PDF analysis an ideal tool for investigating the structure of materials which yield diffraction patterns with large amounts of diffuse scattering between poorly-defined Bragg diffraction peaks such as non-crystalline and poorly ordered materials, like the ones expected for the bicarbonate-rich LCP in solution. Differential PDF analysis of a set of NaHCO_3_ solutions ranging in concentration from 10 mM to 100 mM (containing added NaCl to bring the total Na^+^ concentration to 100 mM in each solution), reveals inter- and intramolecular scattering that can be attributed to the presence of bicarbonate rich LCP. The contribution of the scattering from NaHCO_3_ to the total scattering is isolated by subtraction of PDF patterns of the appropriate concentration NaCl solution (details available in SI). A scattering profile up to 5 Å is for the NaCl control system is shown in the associated content (Supplementary Figure S5). As sodium chloride is replaced by sodium bicarbonate (top to bottom, Figure 3A) differential PDF patterns reveal distinct interactions between sodium and bicarbonate. At a concentration of 40 mM NaHCO_3_ three peaks appear at approximately 2.4, 3.3 and 3.8 Å. The shortest distance is consistent with O-O (G_(O-O)_) and/or Na-O (G_(Na,O1)_) interactions, while the scattering feature at 3.3 Å is consistent with Na-C (G_(Na,C)_) interaction within a sodium-bicarbonate ion association and the feature at 3.8 Å is likely due to Na-O (G_(Na-O2)_, respectively (Sass and Scheuerman, 1962). While, there are several possible explanations for the longest scattering path, the most likely is that it is scattering occurring between Na and an oxygen in carbonate that is proximate to the Na. These peaks exist throughout the transition from NaCl-dominated solution to NaHCO_3_-dominated solution and may be due to ion-pairing and/or association phenomenon. The peaks at 3.3 and 3.8 Å are not present in the nahcolite sample but instead are replaced by a single broad peak at approximately 3.4 Å suggesting that the two interactions are solution state ion interactions. The assigned Na-O peak remains relatively constant in r-distance and in intensity as the solution transitions from being NaCl-dominated to being NaHCO_3_-dominated. At higher sodium bicarbonate concentrations than 40 mM, the Na-O association appears to increase in intensity as well. This association is dissimilar from the much shorter Na-C interaction which occurs in nahcolite. This peak is likely not due to interactions between the sodium ion and the oxygen atom in an associated hydration layer because a similar change in scattering intensity was not observed in the control samples (Supplementary Figure S2) and is not likely due to the onset of solid nahcolite at these highly undersaturated conditions.

PDF analysis is utilized in conjunction with NTA light scattering to detect the ionic behavior accompanying the formation of bicarbonate-rich LCP droplets (Figure 3A). At and above 40 mM NaHCO_3_, PDF analysis detects the emergence of an associations at 2.4, 3.3, and 3.8 Å. This association continues to exist throughout the NaCl→NaHCO_3_ evolution and is believed to be the G_(Na,O)_ and G_(Na, C)_ interactions associated with sodium-to-bicarbonate ion pairing. At 40 mM NaHCO_3_, an association appears at 3.8 Å which, upon increased replacement of NaCl by NaHCO_3_ increases in intensity (Figure 3A). Similarly, NTA detects a large increase in the number of bicarbonate-rich LCP droplets at the same solution conditions (Figure 3B), suggesting that the increase in the amount of long range sodium-bicarbonate ion pairing observed by PDF analysis are due to the formation of bicarbonate-rich LCP as detected by NTA. The concurrent detection of bicarbonate-rich LCP by NTA accompanied by an increase in the fraction of the bicarbonate adopting a long-range sodium-bicarbonate ion pairing as detected by PDF analysis is expected if the bicarbonate ion is a necessary member which results in the formation of bicarbonate-rich LCP.

Evidence from multiple, complimentary analytical techniques supports the coexistence of a bicarbonate-rich LCP phase in equilibrium with a bulk solution bicarbonate phase in simple, undersaturated solutions in the absence of divalent cations. The evidence includes detection of nanoscale size colloids with detectable refractive index contrasts vs. the mother solution when at elevated (>40 mM) bicarbonate concentrations. Additionally, the coexistence of two distinct bicarbonate ion T_2_ relaxations (environments), and the growth in intensity X-ray scattering associated with Na^+^-HCO_3_^-^ ion pair association corresponding to an increase in the number and intensity of scattering events by NTA was reported. The bicarbonate-rich LCP appears to be a solvated solution phase as supported by its refractive index and T_2_ relaxation, which are both consistent with solvated aqueous states. The bicarbonate-rich LCP appears to be stable, due to the large diameter (>50 nm; very large with respect to density fluctuations), long lifetime (at least 6.35 s NMR acquisition time and minutes or more as observed with NTA tracking), and its existence in solutions that are undersaturated with respect to all known solid-phases. At low pH and in the absence of divalent cations, the formation of the bicarbonate-rich LCP is still occurs.

Ion pairing and other strong associations between Ca^2+^ and CO_3_^2-^ have been proposed as a mechanism which leads to a liquid condensed precursor to calcium carbonate through modes of prenucleation clustering (Gebauer and Colfen, 2011). However, the liquid condensed phase identified in this study, does not contain divalent ions, consists of primarily bicarbonate rather than carbonate, and occurs at undersaturated conditions. In the absence of divalent ions (Ca^2+^ and CO_3_^2-^) the transition may be in-part driven by sodium-bicarbonate ion pair affinity. The detection of sodium-bicarbonate ion pairing association correlates with increased bicarbonate-rich LCP scattering detection (Figure 3), suggesting that the observed droplets are indeed bicarbonate-rich and participating in ion pairing with sodium ions. However, it is unlikely that ion pairing/clustering would be the sole driving force for further condensation of additional bicarbonates and sodium ions on a larger scale due to the due to the poor cluster forming ability of monovalent ions beyond a single pairing interaction. An additional mechanism other than those attributable to simple monovalent ion-ion attraction is required to drive the separation, fractionation, and condensation of bicarbonate ions (with sodium ions) into a bicarbonate-rich liquid condensed phase.

One likely candidate is liquid water polymorphism, which is the driving force for aqueous, liquid-liquid phase transitions. According to the principle of liquid water polymorphism, liquid water exists in two or more low energy, amorphous liquid states in equilibrium with each other. These states differ by density and include a high density liquid (HDL) which is directed by distorted, asymmetric hydrogen bonding associations, and a low density liquid (LDL) which is directed by an tetrahedral, symmetrical locally-favored hydrogen bonding associations, both of which are in competition at ambient conditions (Mallamace, 2009; Pettersson and Nilsson, 2015). The attractive and repulsive interactions between like (LDL/LDL, HDL/HDL) and unlike (HDL/LDL) local states of water, respectively, lead to concentration and density fluctuations accounting for many of the anomalous properties of water (Stokely et al., 2010; Overduin and Patey, 2012) and serve as a foundation for liquid-liquid aqueous phase separation (Poole et al., 1995; Franzese et al., 2001). The water arrangements of LDL and HDL exist with an attraction to like-kind but with an antagonism between each other, respectively. Liquid-liquid phase transition without macroscopic separation through this mechanism occurs in a glycerol-water system leading to the emergence of a bi-phasic, aqueous system driven by stabilization of the HDL and LDL local water structures. The phases are distinguished from each other by their relative contrasts in density, composition, and refractive index (Murata and Tanaka, 2012). In parallel, present evidence of density/viscosity, composition, and refractive index contrasts in the sodium bicarbonate/chloride system to suggest that bicarbonate-rich LCP coexists with a bulk solution phase, presumably through the same water polymorphism mechanism of HDL and LDL local water structure stabilization. It is not clear from the presented data whether the bicarbonate-rich LCP separates through a liquid-liquid transition or through a macroscopic liquid-liquid separation, however, other works indicate these liquid-liquid phase transitions occur frequently in aqueous solutions (Mergo and Seto, 2020; Devlin et al., 2023).

**FIGURE 4:**
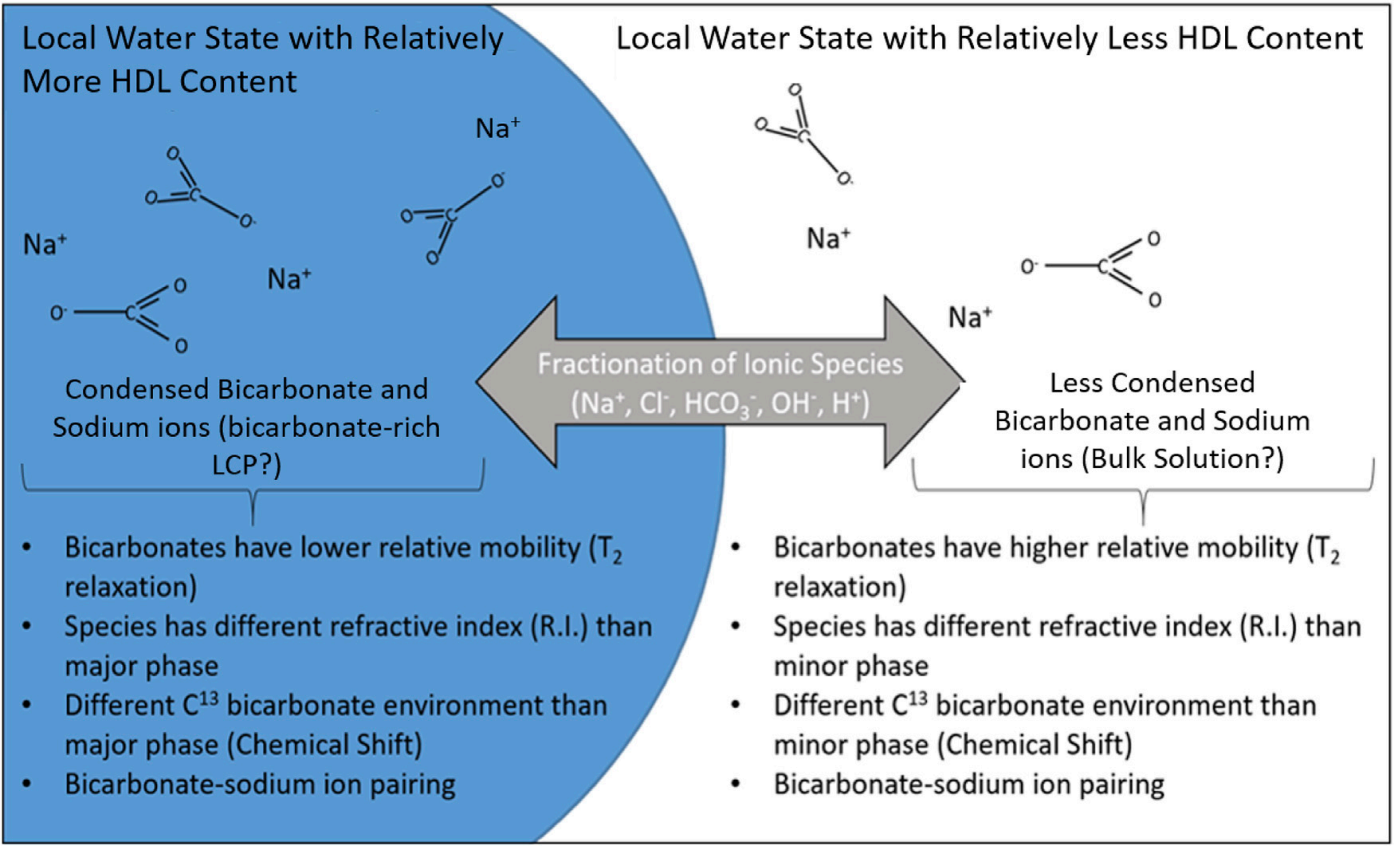
An illustration of a possible motif for bicarbonate LCP formation. The coexistence of both HDL and LDL water structures in solution leads to a fractionation of the solvated ions in solution based on their bias to solvate in each environment. The differences in refractive index, mobility (T_2_ relaxation), and chemical shift may be explained by the fractionation of bicarbonate between the two water structures. The fractionation may be an important mechanism through which systems condense specific ions in close proximity and initiate and direct nucleation of carbonate minerals. These conditions could allow for the coexistence of LDL-rich and HDL-rich phases which, through coalescence, are stabilized into large droplets with the relatively long lifetimes and large size scales observed in this study similar to properties of bicarbonate-rich LCP.

The bias of various ions and solutes to preferentially reside in either the LDL or HDL environments potentially stabilizes the local water state. These stabilized local water states may then coalesce due to the attraction and repulsion between like and unlike local water states (Stokely et al., 2010; Overduin and Patey, 2012). Sodium chloride, which was present in our experiments, has been reported to affect the balance between HDL and LDL in aqueous environments and even facilitates a first-order phase transition. This is due to the ability of alkali metals, such as sodium, to disrupt LDL hydrogen bonding since they prefer hydration in an HDL environment (chloride ions have a negligible preference between the LDL and HDL phases) (Waluyo et al., 2014). It is possible that the bicarbonate oxyanion, perhaps aided by the fractionation preference and charge balance afforded by sodium and chloride ions, fractionates between and stabilizes coexistent separate local water states with different HDL/LDL fractions. Thus allowing for the detection of large regions of density and/or ion concentration contrast (>40 nm diameter) with relatively long lifetimes (seconds to minutes or longer) as measured by multiple techniques (Figures 1–3). This may be one reason why bicarbonate-rich LCP has been difficult to identify to date; in the absence of sufficient alkali metals and bicarbonate to stabilize a separation between local water states with different degrees of HDL and LDL, and sufficient chloride to ensure a charge balance between the two states, the bicarbonate-rich phase may not form or may be too low in concentration to detect. A simple illustration describing how water polyamorphism interacting with ion solvation preference may lead to a similar species as detected here is shown in Figure 4. The designation of the internal aqueous environment of the phases in Figure 4 in terms of relative HDL (“relatively more” vs. “relatively less”) is justified by the fact that bulk water at this temperature (25°C) is dominated by the HDL aqueous structure (Pettersson and Nilsson, 2015) with additional LDL destabilization due to the presence of sodium ions (Nilsson and Pettersson, 2015). The replacement of chloride ions with bicarbonate ions in a solution containing sodium and chloride, as done in this study, may promote a phase transition to occur as ions fractionate between HDL-richer and HDL-poorer local water states depending on their solvation preference and charge balance. This would yield species which resemble the description of bicarbonate-rich LCP to form in coexistence with a bulk solution (Figures 4A,B). This phenomenon may not be specific to the bicarbonate oxyanion system. Other oxyanions (e.g., sulfate and phosphate) display evidence of similar phase transition behavior (You et al., 2013), such as in sulfate systems containing ovalbumin protein (Dumetz et al., 2008) and in aqueous phosphate systems (Franzese et al., 2001) which may form liquid condensed phases through a similar mechanism.

The formation of a naturally occurring, stable liquid condensed phase of bicarbonate in the absence of divalent cations offers a new mechanism to consider when modeling nucleation events. Localized supersaturation that develop during ion condensation and fractionation between the LCP and mother solution phases may partially explain why calcium carbonate nucleation rates are much higher than theoretically expected in the absence of such condensation mechanisms (Vekilov, 2010). LCP formation and persistence also offers an additional mechanism to explain deviation from ideal solute behavior, as commonly accounted for using activity correction coefficients. Many mechanisms, including Debye-Hückel screening, ion-pairing, prenucleation clustering (PNC) (Gebauer et al., 2008) contribute, at least in part, to the changes in activity observed at elevated ionic strength. Bicarbonate-rich LCP is an additional phenomenon that may account for a portion, or even the majority of, the non-ideality of concentrated bicarbonate salt solutions, simply by altering ion activity due to the incorporation of ions into a minor LCP phase.

The existence of a bicarbonate-rich LCP suggests that biomimetic nucleation and precipitation of CaCO_3_ may proceed through both a bicarbonate-dependent pathway, as well as a carbonate-dependent pathway. For example, a fundamental calcium carbonate mineralization reaction is shown in Eq. 1, where CO_2_ is formed along with solid carbonate material via the reaction of Ca^2+^ with two equivalents of HCO_3_^−^.

$${\text{CaCl}}_{2\left(\text{aq}\right)}+2{\text{NaHCO}}_{3\left(\text{aq}\right)}\;\to \;{\text{CaCO}}_{3\left(s\right)}+{\text{CO}}_{2\left(\text{aq}\right)}+H_{2}O_{\left(l\right)}+2{\text{NaCl}}_{\left(\text{aq}\right)}\;\Delta G=-27.3\text{ kJ }{\text{mol}}^{-1}$$ (1)

The calculated Gibb’s free energy assumes standard state temperature and concentrations. Mineralization through reaction one produces one equivalent of CO_2_ for every equivalent of CaCO_3_, which may be evolutionarily desirable, since many organisms that produce large quantities of CaCO_3(s)_ for exoskeletons also require CO_2_ for photosynthesis. In contrast, the conventional mechanism (reaction 2), involves reacting Ca^2+^ with CO_3_^2-^.

$${\text{CaCl}}_{2\left(\text{aq}\right)}+{\text{Na}}_{2}{\text{CO}}_{3\left(\text{aq}\right)}\;\to \;{\text{CaCO}}_{3\left(s\right)}+2{\text{NaCl}}_{\left(\text{aq}\right)}\;\Delta G=-49.87\text{ kJ }{\text{mol}}^{-1}$$ (2)

Maintaining a high pH during mineralization is required for to ensure that carbonate is the predominant form of inorganic carbon in the system for reaction 2. This imposes a metabolic penalty due to the energetic expense of producing or supplying alkalinity. However, biomineralization through reaction one reduces the energetic burden on an organism by favoring bicarbonate utilization, which is prevalent at biologically common pH values (pH 6.5–8.5).

Here, we provide multiple lines of evidence that demonstrate that bicarbonate-rich LCP is not a calcium carbonate nucleation-specific transient phase, but is a stable phase that occurs at equilibrium, in undersaturated conditions and does not require divalent ions to form. Although the question of whether a macroscopic separation is occurring is still open to debate, the data presented here provides direct evidence that the phenomenon of bicarbonate-rich liquid condensed phase (LCP) requires neither supersaturated conditions with respect to a precipitating mineral nor multivalent ions for extensive ion pairing. The driving force for bicarbonate-rich LCP may be due to liquid water polymorphism, which provides a mechanism for the heterogeneous concentration and coalescence of monovalent bicarbonate into a condensed liquid phase. Its existence requires reevaluation of the models involved in nucleation prediction. Explicit accounting for LCP may simplify thermodynamic and kinetic models of electrolyte solutions and provide new insights into previously unexplained observations.

## Data Availability

The original contributions presented in the study are included in the article/Supplementary Material, further inquiries can be directed to the corresponding authors.

## References

[B1] Bewernitz M. A. Gebauer D. Long J. Coelfen H. Gower L. B. (2012a). A metastable liquid precursor phase of calcium carbonate and its interactions with polyaspartate. Faraday Discuss. 159, 291–312. 10.1039/c2fd20080e

[B2] Bewernitz M. A. Gebauer D. Long J. Colfen H. Gower L. B. (2012b). A metastable liquid precursor phase of calcium carbonate and its interactions with polyaspartate. Faraday Discuss. 159, 291–312. 10.1039/c2fd20080e

[B3] Cartwright J. H. Checa A. G. Gale J. D. Gebauer D. Sainz‐Díaz C. I. (2012). Calcium carbonate polyamorphism and its role in biomineralization: how many amorphous calcium carbonates are there? Angew. Chem. Int. Ed. 51 (48), 11960–11970. 10.1002/anie.201203125 23124964

[B4] Devlin S. W. Jamnuch S. Xu Q. Chen A. A. Qian J. Pascal T. A. (2023). Agglomeration drives the reversed fractionation of aqueous carbonate and bicarbonate at the air-water interface. J. Am. Chem. Soc. 145, 22384–22393. 10.1021/jacs.3c05093 37774115

[B5] De Yoreo J. J. Gilbert PUPA Sommerdijk NAJM Penn R. L. Whitelam S. Joester D. (2015). CRYSTAL GROWTH. Crystallization by particle attachment in synthetic, biogenic, and geologic environments. Science. 349 (6247), aaa6760. 10.1126/science.aaa6760 26228157

[B6] Dorvee J. R. Veis A. (2013). Water in the formation of biogenic minerals: peeling away the hydration layers. J. Struct. Biol. 183 (2), 278–303. 10.1016/j.jsb.2013.06.007 23791831 PMC3938164

[B7] Dumetz A. C. Chockla A. M. Kaler E. W. Lenhoff A. M. (2008). Protein phase behavior in aqueous solutions: crystallization, liquid-liquid phase separation, gels, and aggregates. Biophysical J. 94 (2), 570–583. 10.1529/biophysj.107.116152 PMC2157236 18160663

[B8] Egami T. Billinge S. J. L. (2003). Underneath the Bragg peaks structural analysis of Complex materials. Oxford, UK: Pergamon.

[B9] Faatz M. Gröhn F. Wegner G. (2005). Mineralization of calcium carbonate by controlled release of carbonate in aqueous solution. Mater. Sci. Eng. C 25 (2), 153–159. 10.1016/j.msec.2005.01.005

[B10] Filipe V. Hawe A. Jiskoot W. (2010). Critical evaluation of nanoparticle tracking analysis (NTA) by NanoSight for the measurement of nanoparticles and protein aggregates. Pharm. Res. 27 (5), 796–810. 10.1007/s11095-010-0073-2 20204471 PMC2852530

[B11] Franzese G. Malescio G. Skibinsky A. Buldyrev S. V. Stanley H. (2001). Generic mechanism for generating a liquid-liquid phase transition. Nature 409 (6821), 692–695. 10.1038/35055514 11217853

[B12] Gebauer D. Coelfen H. (2011). Prenucleation clusters and non-classical nucleation. Nano Today 6 (6), 564–584. 10.1016/j.nantod.2011.10.005

[B13] Gebauer D. Colfen H. (2011). Prenucleation clusters and non-classical nucleation. Nano Today 6 (6), 564–584. 10.1016/j.nantod.2011.10.005

[B14] Gebauer D. Gunawidjaja P. N. Ko J. Y. P. Bacsik Z. Aziz B. Liu L. (2010). Proto-calcite and proto-vaterite in amorphous calcium carbonates. Angew. Chem. Int. Ed. 49 (47), 8889–8891. 10.1002/anie.201003220 20949576

[B15] Gebauer D. Kellermeier M. Gale J. D. Bergstrom L. Colfen H. (2014). Pre-nucleation clusters as solute precursors in crystallisation. Chem. Soc. Rev. 43 (7), 2348–2371. 10.1039/c3cs60451a 24457316

[B16] Gebauer D. Volkel A. Coelfen H. (2008). Stable prenucleation calcium carbonate clusters. Science 322, 1819–1822. 10.1126/science.1164271 19095936

[B17] Gower L. B. (2008). Biomimetic model systems for investigating the amorphous precursor pathway and its role in biomineralization. Chem. Rev. 108 (11), 4551–4627. 10.1021/cr800443h 19006398 PMC3652400

[B18] Gower L. B. Odom D. J. (2000). Deposition of calcium carbonate films by a polymer-induced liquid-precursor (PILP) process. J. Cryst. Growth 210 (4), 719–734. 10.1016/s0022-0248(99)00749-6

[B19] Lee S. W. Park S. B. Jeong S. K. Lim K. S. Lee S. H. Trachtenberg M. C. (2010). On carbon dioxide storage based on biomineralization strategies. Micron 41, 273–282. 10.1016/j.micron.2009.11.012 20144548

[B20] Mallamace F. (2009). The liquid water polymorphism. Proc. Natl. Acad. Sci. 106 (36), 15097–15098. 10.1073/pnas.0908198106 19805244 PMC2741209

[B21] Menichetti A. Mavridi-Printezi A. Falini G. Besirske P. García-Ruiz J. M. Cölfen H. (2021). Local light-controlled generation of calcium carbonate and barium carbonate biomorphs via photochemical stimulation. Chem. – A Eur. J. 27 (49), 12521–12525. 10.1002/chem.202102321 PMC8456953 34236738

[B22] Mergo J. C. III Seto J. (2020). On simulating the formation of structured, crystalline systems via non-classical pathways. Front. Mater. 7, 75. 10.3389/fmats.2020.00075

[B23] Murata K.-I. Tanaka H. (2012). Liquid–liquid transition without macroscopic phase separation in a water–glycerol mixture. Nat. Mater 11 (5), 436–443. 10.1038/nmat3271 22426459

[B24] Niederberger M. Colfen H. (2006). Oriented attachment and mesocrystals: non-classical crystallization mechanisms based on nanoparticle assembly. Phys. Chem. Chem. Phys. 8 (28), 3271–3287. 10.1039/b604589h 16835675

[B25] Nilsson A. Pettersson L. G. M. (2015). The structural origin of anomalous properties of liquid water. Nat. Commun. 6, 8998. 10.1038/ncomms9998 26643439 PMC4686860

[B26] Overduin S. Patey G. (2012). Understanding the structure factor and isothermal compressibility of ambient water in terms of local structural environments. J. Phys. Chem. B 116 (39), 12014–12020. 10.1021/jp3075749 22963671

[B27] Pettersson L. G. M. Nilsson A. (2015). The structure of water; from ambient to deeply supercooled. J. Non-Cryst Solids 407, 399–417. 10.1016/j.jnoncrysol.2014.08.026

[B28] Poole P. H. Grande T. Sciortino F. Stanley H. E. Angell C. A. (1995). Amorphous polymorphism. Comput. Mater. Sci. 4 (4), 373–382. 10.1016/0927-0256(95)00044-9

[B29] Quan X. Fry E. S. (1995). Empirical equation for the index of refraction of seawater. Appl. Opt. 34, 3477. 10.1364/ao.34.003477 21052163

[B30] Rieger J. Frechen T. Cox G. Heckmann W. Schmidt C. Thieme J. (2007). Precursor structures in the crystallization/precipitation processes of CaCO3 and control of particle formation by polyelectrolytes. Faraday Discuss. 136 (0), 265–277. 10.1039/b701450c 17955814

[B31] Sass R. L. Scheuerman R. F. (1962). The crystal structure of sodium bicarbonate. Acta Crystallogr. 15 (1), 77–81. 10.1107/s0365110x62000158

[B32] Schenk A. S. Zope H. Kim Y.-Y. Kros A. Sommerdijk NAJM Meldrum F. C. (2012). Polymer-induced liquid precursor (PILP) phases of calcium carbonate formed in the presence of synthetic acidic polypeptides-relevance to biomineralization. Faraday Discuss. 159 (0), 327–344. 10.1039/c2fd20063e

[B33] Sebastiani F. Wolf S. L. P. Born B. Luong T. Q. Cölfen H. Gebauer D. (2016). Water dynamics from THz spectroscopy reveal the locus of a liquid–liquid binodal limit in aqueous CaCO3 solutions. Angew. Chem. Int. Ed. 56, 490–495. 10.1002/anie.201610554 28029201

[B34] Stokely K. Mazza M. G. Stanley H. E. Franzese G. (2010). Effect of hydrogen bond cooperativity on the behavior of water. Proc. Natl. Acad. Sci. 107 (4), 1301–1306. 10.1073/pnas.0912756107 20080604 PMC2824356

[B35] Vekilov P. G. (2010). Nucleation. Cryst. Growth and Des. 10 (12), 5007–5019. 10.1021/cg1011633 PMC2995260 21132117

[B36] Wallace A. F. Hedges L. O. Fernandez-Martinez A. Raiteri P. Gale J. D. Waychunas G. A. (2013). Microscopic evidence for liquid-liquid separation in supersaturated CaCO3 solutions. Science. 341 (6148), 885–889. 10.1126/science.1230915 23970697

[B37] Waluyo I. Nordlund D. Bergmann U. Schlesinger D. Pettersson L. G. M. Nilsson A. (2014). A different view of structure-making and structure-breaking in alkali halide aqueous solutions through x-ray absorption spectroscopy. J. Chem. Phys. 140 (24), 244506. 10.1063/1.4881600 24985653

[B38] Wolf S. E. Böhm C. Harris J. Hajir M. Mondeshki M. Marin F. (2015). “Single nanogranules preserve intracrystalline amorphicity in biominerals,” Key engineering materials (Trans Tech Publications).

[B39] Wolf S. E. Gower L. B. (2017). “Challenges and perspectives of the polymer-induced liquid-precursor process: the pathway from liquid-condensed mineral precursors to mesocrystalline products,” in New perspectives on mineral nucleation and growth: from solution precursors to solid materials. Editors Van Driessche A. E. S. Kellermeier M. Benning L. G. Gebauer D. (Cham: Springer International Publishing), 43–75.

[B40] Wolf S. E. Leiterer J. Kappl M. Emmerling F. Tremel W. (2008). Early homogenous amorphous precursor stages of calcium carbonate and subsequent crystal growth in levitated droplets. J. Am. Chem. Soc. 130 (37), 12342–12347. 10.1021/ja800984y 18717561

[B41] Wolf S. E. Leiterer J. Pipich V. Barrea R. Emmerling F. Tremel W. (2011a). Strong stabilization of amorphous calcium carbonate emulsion by ovalbumin: gaining insight into the mechanism of 'polymer-induced liquid precursor' processes. J. Am. Chem. Soc. 133 (32), 12642–12649. 10.1021/ja202622g 21736300 PMC3170880

[B42] Wolf S. E. Lieberwirth I. Natalio F. Bardeau J. F. Delorme N. Emmerling F. (2012). Merging models of biomineralisation with concepts of nonclassical crystallisation: is a liquid amorphous precursor involved in the formation of the prismatic layer of the Mediterranean Fan Mussel Pinna nobilis? Faraday Discuss. 159, 433–448. 10.1039/c2fd20045g

[B43] Wolf S. E. Muller L. Barrea R. Kampf C. J. Leiterer J. Panne U. (2011b). Carbonate-coordinated metal complexes precede the formation of liquid amorphous mineral emulsions of divalent metal carbonates. Nanoscale 3 (3), 1158–1165. 10.1039/c0nr00761g 21218241 PMC3111071

[B44] You Y. Renbaum-Wolff L. Bertram A. K. (2013). Liquid–liquid phase separation in particles containing organics mixed with ammonium sulfate, ammonium bisulfate, ammonium nitrate or sodium chloride. Atmos. Chem. Phys. 13 (23), 11723–11734. 10.5194/acp-13-11723-2013

