# Supplementary material for: A bicarbonate-rich liquid condensed phase in non-saturated solutions in the absence of divalent cations
Source: Front Bioeng Biotechnol. 2024 Apr 30;12:1382047. doi: 10.3389/fbioe.2024.1382047 (PMC11091711; doi:10.3389/fbioe.2024.1382047)
Supplement: Supplementary file 1 [file DataSheet1.pdf]

Associated Content: A Bicarbonate-Rich Liquid Condensed Phase in non-saturated solutions in the Absence of Divalent Cations

Mark A. Bewernitz,[1*] Matthew Ginder-Vogel,[2] Stephan E. Wolf,[3,4] Jong Seto,[5] Brent R. Constantz,[1,6*]

[1]Physical Sciences Department, College of Arts and Sciences, Embry-Riddle Aeronautical University, Daytona Beach, Fl 32114†

[2]Environmental Chemistry and Technology Program, Department of Civil and Environmental Engineering, University of Wisconsin – Madison, Madison, WI 53706

[3]Institute of Glass and Ceramics (WW3), Department of Materials Science and Engineering, Friedrich-Alexander-University Erlangen-Nürnberg, Martensstrasse 5, 91058 Erlangen, Germany

[4]Interdisciplinary Center for Functional Particle Systems (FPS), Friedrich-Alexander University Erlangen-Nürnberg (FAU), Haberstrasse 9a, 91058 Erlangen, Germany

[5]Center for Biological Physics and School for Engineering of Mater, Transport, and Energy, Arizona State University, Tempe, AZ 85287

[6]Geological and Environmental Sciences, Stanford University, Stanford, CA 94305

ABSTRACT: Bicarbonate (HCO_3^-) and sodium (Na^+)-containing solutions contain droplets of a separate, bicarbonate-rich liquid condensed phase (LCP) that have higher concentrations of HCO_3^- relative to the bulk solution in which they reside. The existence and composition of the LCP droplets has been investigated by nanoparticle tracking analysis, nuclear magnetic resonance spectroscopy, refractive index measurements and X-ray pair distribution function analysis. The bicarbonate-rich LCP species is a previously unaccounted-for, ionic phenomenon which occurs even in solutions with solely monovalent cations. Its existence requires re-evaluation of models used to describe and model aqueous solution physicochemistry, especially those used to describe and model carbonate mineral formation.

NTA Image Collection: Data was collected on a NS-500 nanoparticle tracking analyzer (Malvern). Raw data consisted of 60-second recordings (in triplicate if error bar is present in data) of the scattering projections of various solutions at a camera length setting of 15. Still shots used in figures are representative still shots of each recording. The recording was processed to generate the histograms of particle size vs. particle count with a bin size of 20 nm. The settings used to process the raw data included a detection threshold of 6, auto-blur, auto-minimum expected particle size, and a solution viscosity equal to that of water at 22 °C (0.96 cP) for data presented in Figure 1 and 25 °C (0.89cP) for data represented in Figure 3 and Figure S1.

Sample Preparation for NTA Image Collection: The simple bicarbonate solution seen in Figure 1A consisted of 50 mM $NaHCO_3$, (Sigma-Aldrich, 144-55-8) and 50 mM NaCl (Sigma-Aldrich, S9888). The control with which it was compared in Figure S2 consisted of a 100 mM NaCl solution. All samples other than the naturally occurring seawater were created using deionized nanopure water with these salt additives. All samples were filtered prior to analysis with a 20 nm diameter pore size syringe filter (Whatman Cat. No. 6809-1002).

Sample Preparation for NMR Measurements: A solution containing 100 mM $NaHCO_3$ and 100 mM NaCl was created using 100% ^{13}C substituted $NaHCO_3$ (Cambridge Isotopes, 372382). No chemical shift standard was added to the solution to ensure electrolyte behavior was naturally occurring. Samples were filtered through a syringe filter of pore diameter 20 nm (Whatman, 6809-1002). All NMR data was obtained using a Varian Inova 500 magnet operating at 126 MHz using a 5 mm broadband probe. All experiments were conducted at 298 Kelvin. Deuterium oxide (Aldrich, 151882) was used to obtain a lock at a volume fraction of 2.5% of the total sample. Data was processed using NUTS™ and Microsoft Excel software when deconvolution of overlapping spectral peaks was required. 90° pulses were used with acquisition times of 5.62 seconds. The T_2 relaxation measurements were conducted using a Carr-Purcell-Meibloom-Gill (CPMG) sequence with increasing tau (τ) times of 0.025, 0.05, 0.1, 0.2, 0.4, 0.8, 1.6, 3.2, 6.4 seconds. Four scans were averaged for each tau time. Each spectrum contained 16,385 points, at a 1256 Hz sweep width, with a 5.62 second acquisition time which yields a digital resolution of 0.15 Hz/point.

Sample Preparation and Approach to Refractive Index Approximation: Species in the size range of the bicarbonate-rich LCP are generally in the Rayleigh scattering regime, where a close approximation of the efficiency of light scattering is given by equation (S1) below:

$$I \propto \sigma_s = \frac{2\pi^5}{3}\frac{d^6}{\lambda^4}\left(\frac{n^2-1}{n^2+2}\right)^2 \quad (1)$$

Where:

I is the measured intensity of the scattering event

σ_s is the scattering cross section

d is the species diameter

λ is the wavelength of incident light, here λ = 402 nm

n is the ratio of the scattering species refractive index to the solvent refractive index

Equation (S1) relates the measured intensity of a scattering event (I) to the diameter of the scattering center species (d), the wavelength of incident light (λ) and the refractive indices (RI) of the scattering species and solvent. The NTA technique measures I directly and d by means of Brownian motion. If the RI of the solvent is known, a standard with a known RI can be used to calibrate the technique to account for software and equipment measurements of the relative intensity. Equation (S1) can then be used to calculate a first approach to the RI for the scattering species (1, 2).

To determine the RI of bicarbonate-rich LCP, silica (SiO_2) nanoparticles of 50, 80, 100 nm (hydrated diameters of 62, 92, 116 nm, respectively) were used as the standard reference material (RI = 1.51). Equal masses of the different sized SiO_2 nanoparticles were placed into a solution of 100 mM Na_2CO_3 to the concentration of approximately 10^8 particles/ml. The standard solution was then titrated with 1 N HCl, to pH 9.0, and analyzed for light scattering events using NTA. Figure S5 displays the standard curve. This standard curve was applied to the data shown in Figure 2C to demonstrate the reliability of the technique and to verify and validate the technique.

Data points represent scattering events tracked for more than at 20 random walk events were included in our analysis. At 25 frames/sec that is 0.8 seconds of minimum tracking time. The maximum tracking time was the duration of the analysis of 30 seconds to a minute (some species were tracked for the duration). Species tracked for longer time periods (more random walk frames) are weighted more heavily in our qualitative analysis than scattering events tracked for lesser random walk frames. The size of the data point assigned is qualitatively proportional to the amount of random walk frames; between the minimum and the maximum. SiO_2 nanoparticles were used as a calibration standard. Raleigh intensity vs. diameter profile consistent with an RI = 1.475 for SiO_2 (3) was visually fit to encompass the SiO_2 scattering (red line, shaded red area). Using this standard, the refractive index of the standard curve was adjusted to yield Raleigh curves that overlap with LCP scattering data points. Many overlapping, highly relevant LCP scattering events (green shaded region) have a refractive index of ~1.355 ("reasonable fit" line) due to the many large data points. Additional data points outside of the "reasonable fit" regime are encompassed by a "lower limit" RI of ~1.347 (black line).

Sample Preparation for Pair Distribution Function (PDF) Analysis: Stock solutions of 100 mM $NaHCO_3$ or 100 mM NaCl were prepared in volumetric glassware, using ACS grade chemicals, 12 hours prior to analysis at Argonne National Lab. Solutions were prepared by adding the appropriate amount salt to the volumetric flask and Milli-Q water (18 megaohm) was added to obtain a final volume of 100 mL. These solutions were stored in zero-headspace, sealed, glass vials until sample preparation.

Samples for analysis using high energy X-ray scattering were prepared from the 100 mM $NaHCO_3$ and 100 mM NaCl solutions within 2 hours of analysis. Samples were prepared by addition of the appropriate amount of each solution to achieve a final volume of 10 mL and a $NaHCO_3$ concentration of 0, 10, 20, 30, 40, 50, 60, 70, 80, 90 or 100 mM and NaCl concentrations of 100, 90, 80, 70, 60, 50, 40, 30, 20, 10 or 0 This ensured that the total Na^+ concentration remained constant at 100 mM throughout all experiments. Samples were immediately filtered using a 0.020 nm syringe filter into a fresh, acid-washed scintillation vial. Following filtration, a small amount sample was loaded into a Kapton tube with a 2 mM diameter for data collection using a syringe and needle.

X-ray scattering measurements were performed at beamline 11-ID-B at the Advanced Photon Source, which is dedicated to Pair-Distribution-Function measurements with area detectors. The instrument operates at 58.66 keV. Liquids were loaded into Kapton tubing with an inner diameter of ~ 2mm. The measurements were performed using the rapid-acquisition PDF technique (4), employing a Perkin Elmer amorphous silicon detector which was mounted orthogonally to the beam path. The image plate was exposed for 10 s and this was repeated 300 times for a total collection time of 3,000 s for each sample. Diffraction data on empty polyimide tubing was also collected for background removal in the PDF data reduction.

Fit2D (5) was used to integrate and convert 2-D data into 1-D intensity versus wave vector (Q) data. The sample-to-detector distance, beam center position and tilt angles of the detector, relative to the beam path, were calibrated using a CeO_2 standard.

One major challenge in processing this data is to isolate the scattering contribution from the LCP and aqueous carbonate from the scattering contribution of the background electrolyte solution. In order to isolate the scattering of the LCP and the dissolved carbonate the scattering pattern of each NaCl solution was subtracted from the appropriate solution containing NaCl and $NaHCO_3$. For example, the scattering pattern for the 40 mM NaCl solution was subtracted from the scattering pattern for the solution containing 40 mM NaCl and 60 mM $NaHCO_3$. In

the case of the sample containing 100 mM $NaHCO_3$ the scattering pattern for DDI water was used in the background subtraction. The background pattern was scaled to maintain an absolute difference of 500 cps at 20 degrees 2-theta.

Data Reduction for Pair Distribution Function (PDF) Analysis: The PDF, G(r), for a finite scattering body is defined as follows (5, 6):

$$G(r) = 4\pi r \gamma_0(r)[\rho_{\text{bulk}}(r) - \rho_0]$$

where $\rho_{\text{bulk}}(r)$ and ρo are the local and average atomic number densities of an infinite body of the same internal structure as the finite body, respectively, G(r) accounts for the finite size and shape of the body and *r* is the radial distance. The PDF has peaks at characteristic distances separating pairs of atoms, and thus reflects the atomic structure.

The PDF is the Fourier transform of the experimentally observable total structure function (6, 7) S(Q), i.e.

$$G(r) = \frac{2}{\pi} \int_{Q\text{min}}^{Q\text{max}} F(Q) \sin(Qr) \mathrm{d}Q$$

$$F(Q) = Q[S(Q) - 1]$$

where F(Q) is the reduced structure function, Q is the magnitude of the wave vector where $Q = \frac{4\pi \ \sin\theta}{\lambda}$, 2θ is the angle between the incoming and outgoing radiation beams, and k is the wavelength of the X-rays used. The structure function is related to the coherent part of the total diffraction intensity of the material as follows (7):

$$S(Q) = 1 + \frac{[I^{\text{coh}}(Q) - (\sum c_{\text{i}} \mid f_{\text{i}}(Q)|^2)]}{|\sum C_{\text{i}} F_{\text{i}}(Q)|^2}$$

where $I^{coh}(Q)$ is the coherent scattering intensity per atom in electron units and ci and fi are the composition of the atomic species of type i and its X-ray scattering form factor, respectively.

In the Fourier transform step to get G(r) from S(Q) the data are truncated at a finite minimum, Qmin and maximum, Qmax values of the wave vector, Q. Here, Qmax was optimized to avoid large termination effects while minimizing the statistical noise coming from the high-Q region where the signal–noise ratio becomes unfavorable. A Qmax = 25.0 $Å^{-1}$ was found to be optimal for the aqueous samples. The total scattering structure function, S(Q), and G(r) were obtained using PDFgetX2 (8), where standard corrections (7) were applied as well as those unique to the area detector geometry (4).

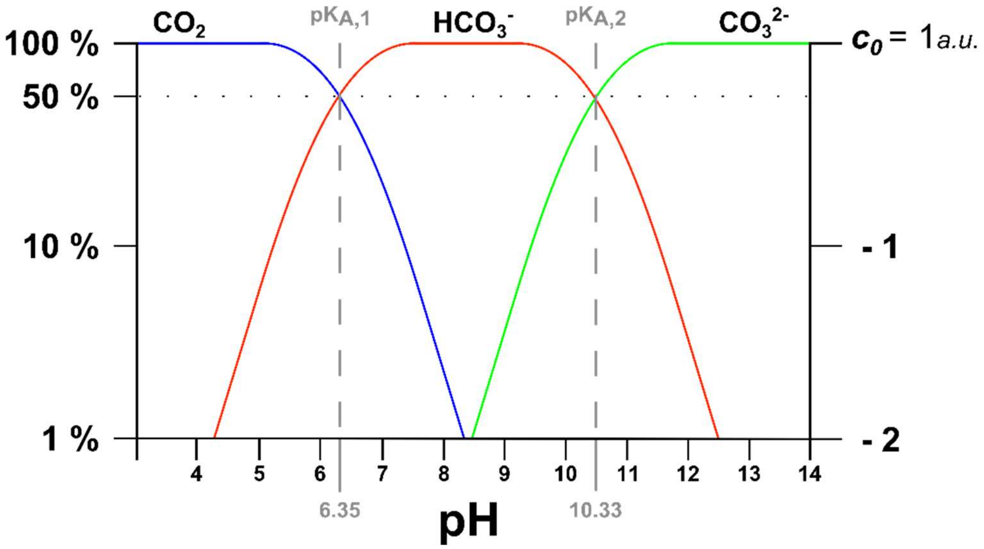

Fig. S1. The Haag digram of the carbonate system vs pH. At pH values of 10 or lower, the carbonate ion becomes less favorable with respect to the bicarbonate ion. Between the pH values of 9 and 7, the bicarbonate ion is the dominant form of the carbonate ion in solution. This pH range is more representative of biological systems and seawater conditions as a whole than pH values above the second pKa (10.33). Many studies attribute mechanisms and behaviors to calcium carbonate nucleation and growth at pH values above the second pKa where the carbonate ion is dominant. Such studies may not detect the role that the bicarbonate may play in the non-classical nucleation and growth of calcium carbonate through the formation of a liquid condensed phase (LCP).

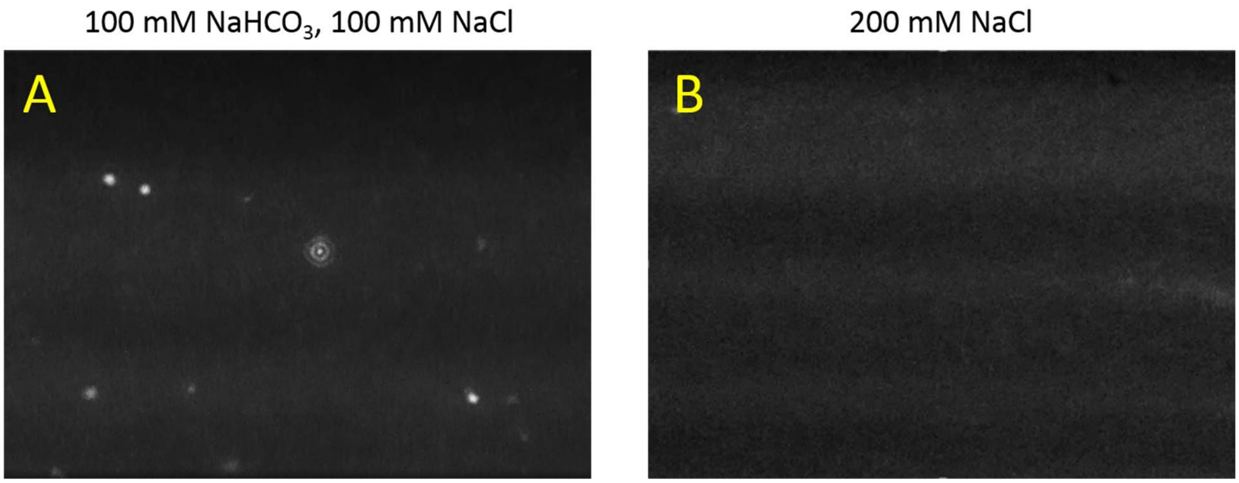

Fig. S2. Bicarbonate ions form bicarbonate-rich liquid condensed phase. Still-shots of the scattering projections obtained by nanoparticle tracking analysis (NTA) strongly suggest that the bicarbonate ion participates in a condensation, as reported for bicarbonate-rich LCP. A) A solution containing 100 mM NaHCO3 and 100 mM NaCl contains many scattering events due to the formation of bicarbonate-rich LCP. B) A solution containing 200 mM NaCl does not display scattering events at the same conditions. This is evidence that the bicarbonate ion participates in a condensation to form bicarbonate-rich LCP even in relatively simple, undersaturated solutions.

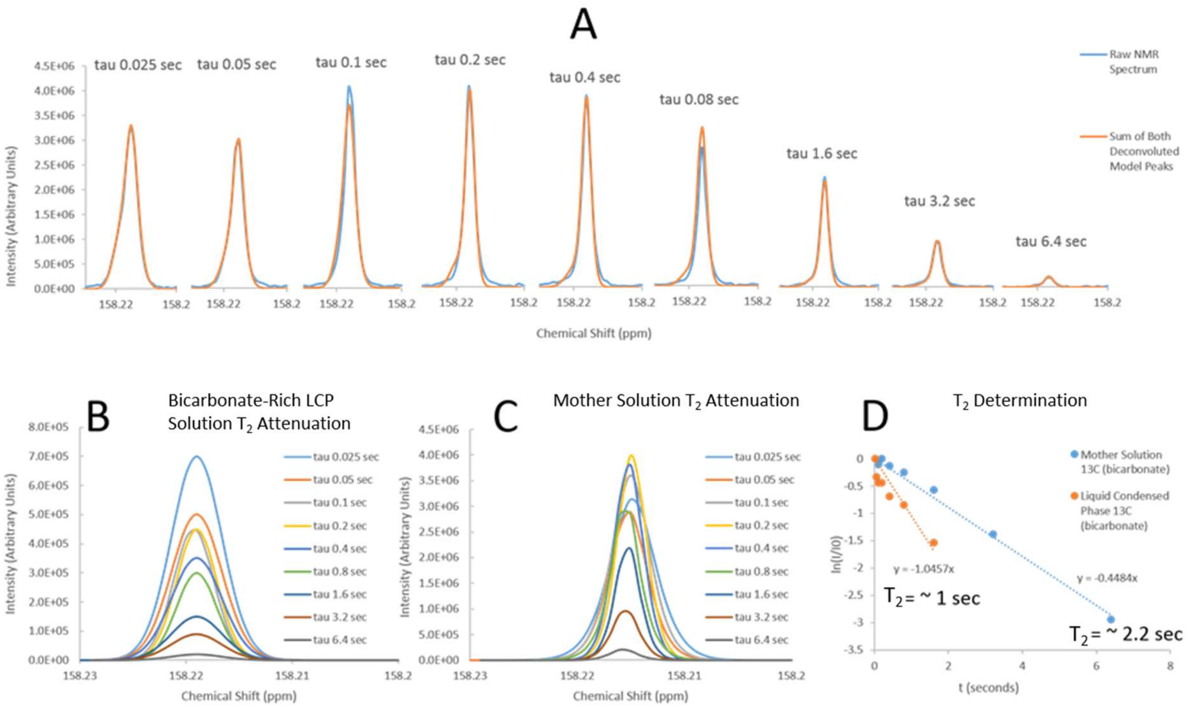

Fig. S3. The determination of the t2 values of bicarbonate-rich LCP and mother solution by deconvolution of a CPMG NMR sequence. A) The T_2 "slices" of a CPMG experiment on a 100 mM $NaHCO_3$ (100%^{13}C enriched) and 100 mM NaCl solution. The ^{13}C spectra at various tau times were deconvoluted using two Gaussian model curves. The sum of the curves is shown in orange and is a reasonable fit to the experimental data shown in blue. B) The attenuation of the deconvoluted bicarbonate-rich LCP curves as tau is increased. C) The attenuation of the deconvoluted mother solution curve as tau is increased. D) A plot of the intensity attenuation of the LCP and mother solution curves as a function of time. The slope of the curve is $1/T_2$. The significant difference between the T_2 relaxations of the two deconvoluted curves suggests that the bicarbonate ion is experiencing two distinct chemical environments (phases) which are solution state phases (large T_2 relaxation times).

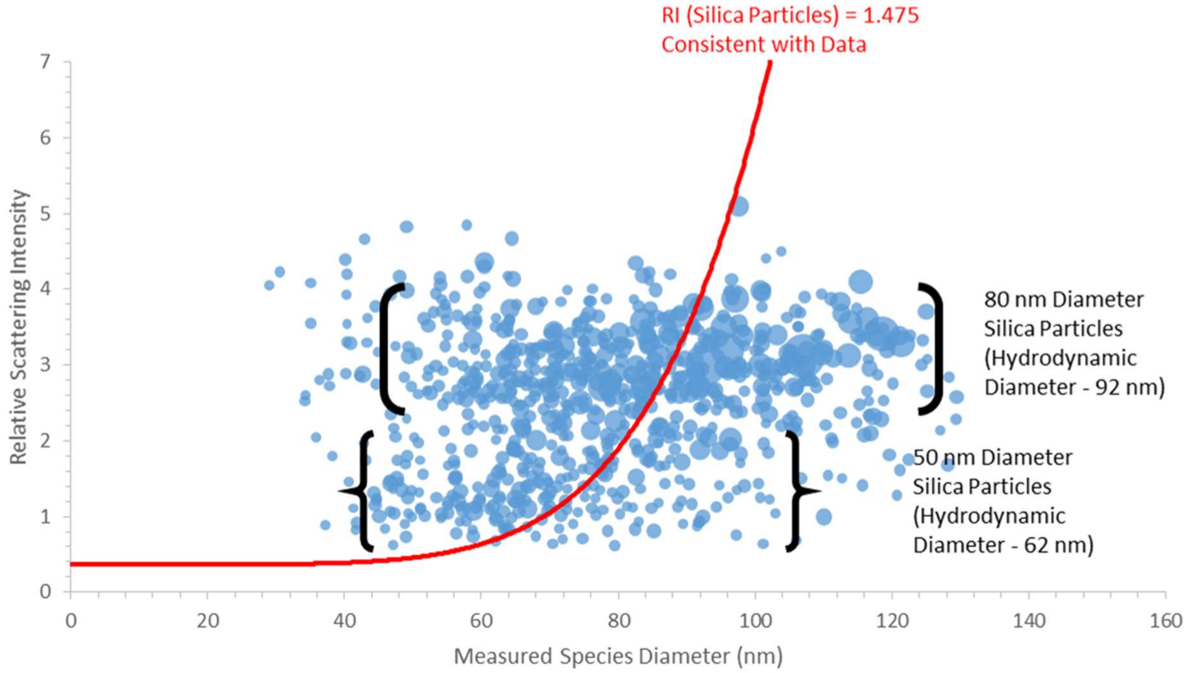

Fig. S4. The standardization of nanoparticle tracking analysis with silica particles for refractive index measurements. Scattering intensity vs. diameter of silica nanoparticles in water obtained from the NS500 nanoparticle tracking analyzer. To establish a standard curve and calibrate the NS500 NTA for refractive indices measurements, silica (SiO_2) nanoparticles (nano-Composix) of 50, and 80 nm (hydrated diameters of 62, and 92 nm, respectively) were used as the standard reference material (RI = 1.51). A curve was qualitatively fitted using the Raleigh approximation (equation S1) to fit the intensity of the scattering events with the measured size of the particles. Small data points (small blue circles) represent scattering events whose random walk was tracked for approximately 1-3 seconds (25 to 75 random walks). Large data points (larger blue circles) represent scattering events whose random walk was tracked for approximately > 3 seconds with circle size proportional to the amount of time tracked, yielding a more certain intensity and size estimate. The area of the data points (blue circles) represent the relative statistical certainty of the measurement.

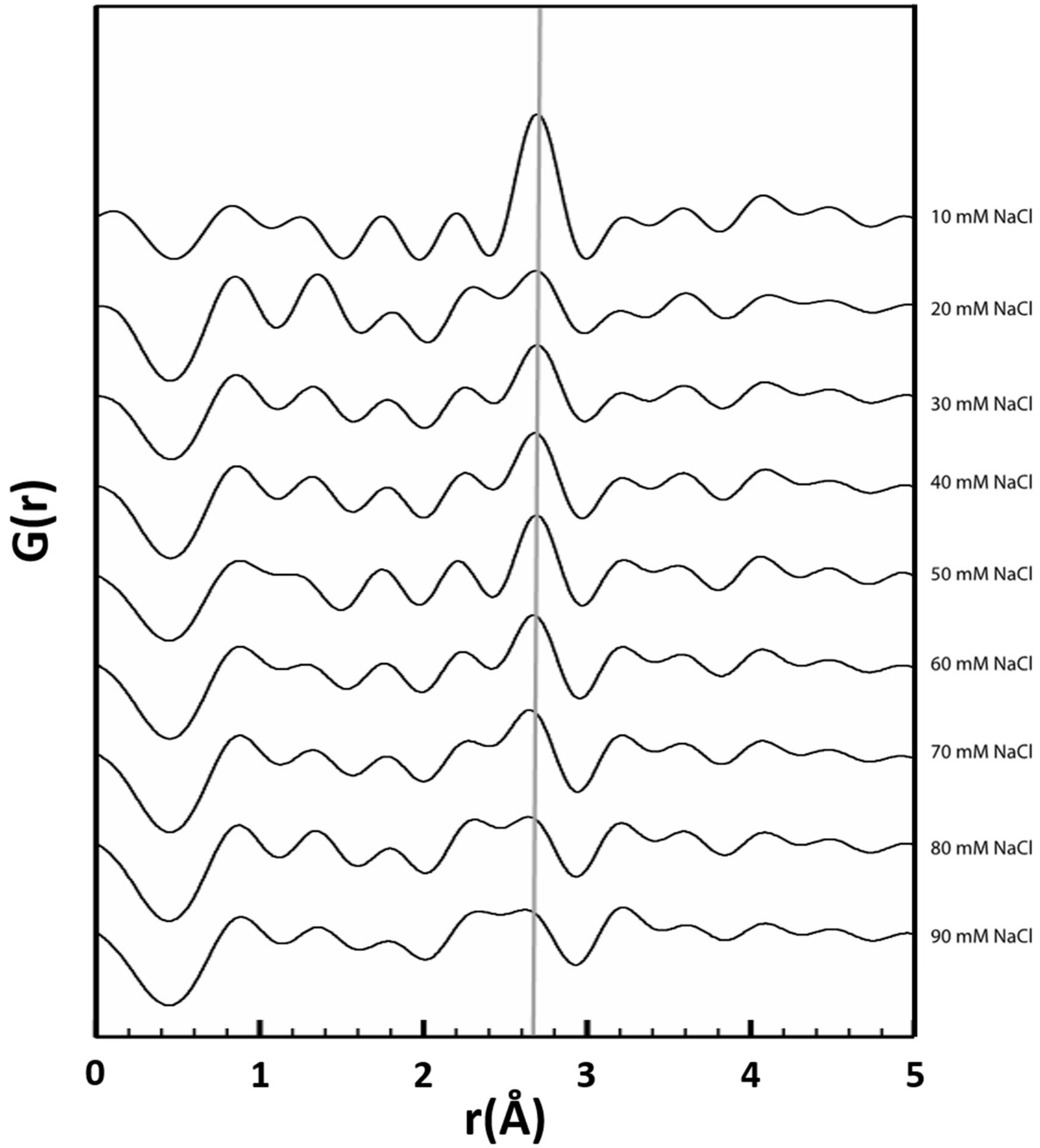

Fig. S5. Pair Distribution Function (PDF) of various sodium chloride concentrations. The PDF of the water and sodium chloride solutions were subtracted as background functions from the experimental bicarbonate mixtures to isolate the PDF associated with sodium bicarbonate. The peak observed at r = 2.8 is the $G_{(O,O)}$ in between water molecules.

REFERENCES

1. Gardiner C, Ferreira YJ, Dragovic RA, Redman CW, Sargent IL. Extracellular vesicle sizing and enumeration by nanoparticle tracking analysis. Journal of extracellular vesicles. 2013;2(1):19671.
2. Gardiner C, Shaw M, Hole P, Smith J, Tannetta D, Redman CW, Sargent IL. Measurement of refractive index by nanoparticle tracking analysis reveals heterogeneity in extracellular vesicles. Journal of extracellular vesicles. 2014;3(1):25361.
3. Khlebtsov BN, Khanadeev VA, Khlebtsov NG. Determination of the size, concentration, and refractive index of silica nanoparticles from turbidity spectra. Langmuir. 2008;24(16):8964-70.
4. Chupas PJ, Qiu X, Hanson JC, Lee PL, Grey CP, Billinge SJL. Rapid-acquisition pair

distribution function (RA-PDF) analysis. Journal of Applied Crystallography. 2003;36(6):1342-7.
5. Hammersley A. FIT2D: an introduction and overview. European Synchrotron Radiation Facility Internal Report ESRF97HA02T. 1997.
6. Farrow CL, Billinge SJL. Relationship between the atomic pair distribution function and small-angle scattering: implications for modeling of nanoparticles. Acta Crystallographica Section A. 2009;65(3):232-9.
7. Egami T, Billinge SJL. Underneath the Bragg Peaks Structural Analysis of Complex Materials. Oxford, UK: Pergamon; 2003.
8. Qiu X, Thompson JW, Billinge SJ. PDFgetX2: a GUI-driven program to obtain the pair distribution function from X-ray powder diffraction data. Journal of Applied Crystallography. 2004;37(4):678-.
